# Endovascular treatment of a right pulmonary sequestration supplied by an aneurysmal aberrant artery originating from the abdominal aorta

**DOI:** 10.1590/1677-5449.201901602

**Published:** 2022-05-23

**Authors:** Leopoldo Marine, Jose Ignacio Torrealba, Francisco Valdes, Renato Mertens, Francisco Vargas, Michel Bergoeing, Daniel Vallejos

**Affiliations:** 1 Pontificia Universidad Católica de Chile, Escuela de Medicina, Santiago, Chile.

**Keywords:** bronchopulmonary sequestration, endovascular procedures, embolization, therapeutic, sequestro broncopulmonar, procedimentos endovasculares, embolização terapêutica

## Abstract

Endovascular embolization of arteries feeding pulmonary sequestrations is a growing therapeutic option. A 51-year-old woman with chest pain and hemoptysis was admitted. During hospitalization she presented 150 mL hemoptysis, hypotension, and hematocrit fell to 23.3%. Contrast-enhanced computed tomography confirmed a pulmonary sequestration irrigated by an aneurysmal artery from the abdominal aorta. The patient underwent endovascular coil embolization of the artery feeding the aneurysm and an Amplatzer device was deployed in the proximal third of the sequestration artery. Subsequent contrast-enhanced computed tomography confirmed complete thrombosis of the aberrant artery feeding the aneurysm and absence of irrigation of the pulmonary sequestration. At 56 months follow-up the patient remains asymptomatic, tomography showed involution of the sequestration and complete thrombosis of the aberrant artery. The challenges presented by the different treatment alternatives are discussed.

## Resumo

A embolização endovascular das artérias que alimentam os sequestros pulmonares é uma opção terapêutica em crescimento. Uma mulher de 51 anos com dor torácica e hemoptise foi internada. Durante a internação, ela apresentou hemoptise de 150 mL, hipotensão e queda do hematócrito para 23,3%. A tomografia computadorizada com contraste confirmou um sequestro pulmonar irrigado por uma artéria aneurismática originária da aorta abdominal. A paciente foi submetida a embolização endovascular da artéria que alimentava o aneurisma com uso de coils e dispositivo Amplatzer no terço proximal da artéria sequestrante. A tomografia subsequente confirmou a trombose completa da artéria aberrante que alimentava o aneurisma e a ausência de irrigação dentro do sequestro pulmonar. No seguimento de 56 meses, a paciente permanecia assintomática, e a tomografia mostrou involução do sequestro e trombose completa da artéria aberrante. Os desafios apresentados pelas diferentes alternativas de tratamento são discutidos neste artigo.

## INTRODUCTION

Pulmonary sequestration (PS) is a rare congenital malformation that accounts for less than 6.5% of all pulmonary malformations^1^^,^^2^ and most frequently affects the left lower lobe.^3^ It is defined as nonfunctional lung tissue without tracheobronchial communication that is supplied by an anomalous systemic artery.

Patients may be asymptomatic, but potential complications such as recurrent pulmonary infections, congestive heart failure, and massive hemoptysis make it desirable to treat all patients once diagnosed.

Conventional treatment for PS is open surgical resection via posterolateral thoracotomy. In recent years, less invasive interventions have been introduced including video-assisted thoracoscopic surgery (VATS),^3^ endovascular interventions, or a combination of these. Endovascular embolization with coils or plugs is frequently used and coverage of the origin of the aberrant aneurysmal artery with an endograft has also been described.^4^

We present a clinical case of symptomatic PS with severe hemoptysis associated with an aneurysmal feeding artery arising from the abdominal aorta, the therapeutic challenge involved, and the treatment performed, together with a review of the literature. The Research Ethics Committee approved this study (decision number ID 210727004).

### Part I - Clinical situation

A 51-year-old otherwise healthy woman, with no history of congenital or pediatric disease, was admitted to the hospital after two days of cough, dyspnea, chest pain, and hemoptysis. The patient was a non-smoker and had no history of drug abuse, previous exposure to air pollutants, or presence of trauma or seasonal illness. She denied NSAID use, fever or weight loss. Her ER blood pressure was 85/54 mm Hg with a heart rate of 123 bpm and oxygen saturation of 88%. Physical examination was positive for pallor, tachypnea, tachycardia, and bilateral crepitations and rhonchi on auscultation, worse in the right lower lung field.

**Figure 1:**
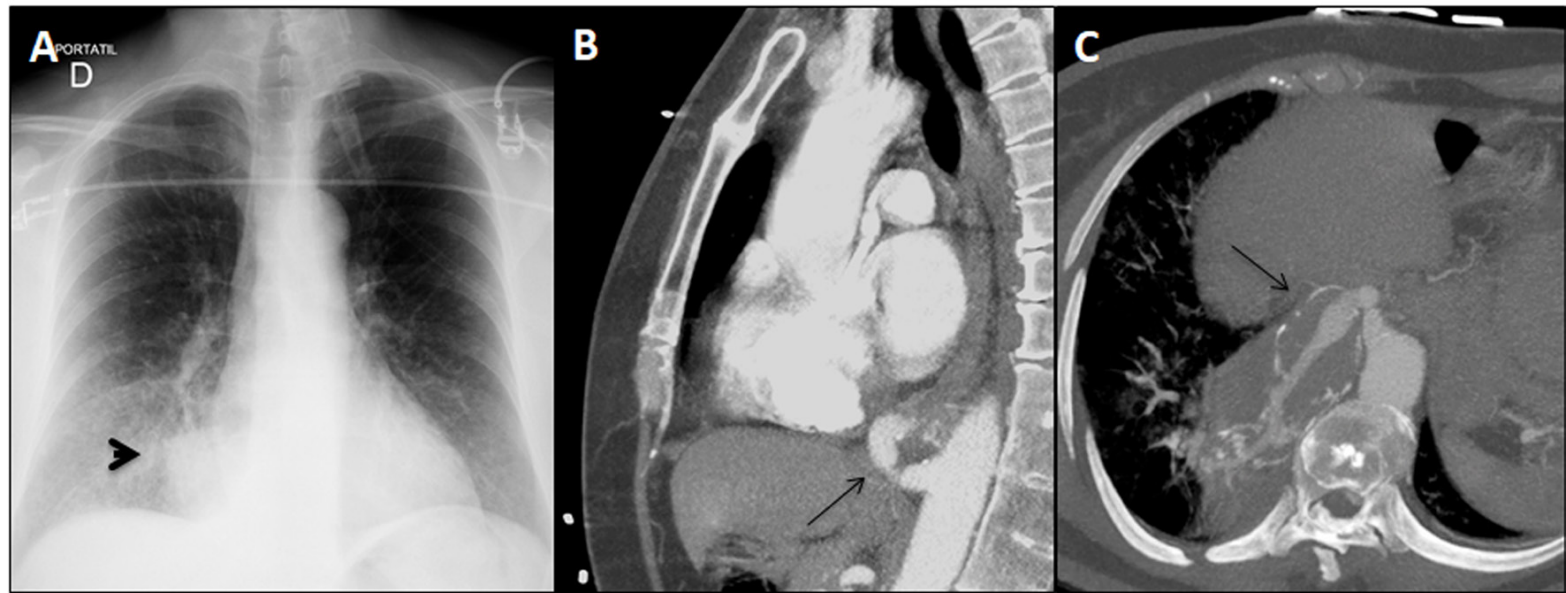
Initial imaging tests. (A) Parenchymal opacity on Chest X-ray (arrowhead); (B) and (C) Origin of aberrant aneurysmal artery with calcified wall and mural thrombus supplying abnormal pulmonary parenchyma on CTA (black arrows).

Admission laboratory tests were normal, with 35.0% hematocrit. Chest X-ray showed a parenchymal opacity at the base of the right hemithorax (Figure 1A), suggestive of acute pneumopathy or intra-alveolar hemorrhage. Initial treatment consisted of volume resuscitation and 30% oxygen with mask. During hospitalization she presented an episode of hemoptysis of 150 ml and hematocrit fell to 23.3%. Upper endoscopy was normal.

A contrast-enhanced computed tomography (CTA) of the chest, abdomen, and pelvis revealed increased pulmonary parenchymal density with ground-glass infiltrates within the right medial and posterior basal lower lobe containing multiple cysts that were irrigated by a tortuous and aneurysmal artery originating from the abdominal aorta just above the celiac trunk; all findings suggestive of PS. The feeding artery measured 9 mm at its origin in the abdomen (Figure 1B) and followed a transverse and ascending course toward the abnormal pulmonary parenchyma in the right basal lobe. It became aneurysmal (33 × 32 × 74 mm), with mural calcifications, atherosclerotic changes, and partial thrombosis (Figure 1C). There was also a mild right pleural effusion.

### Part II – What was done

**Figure 2:**
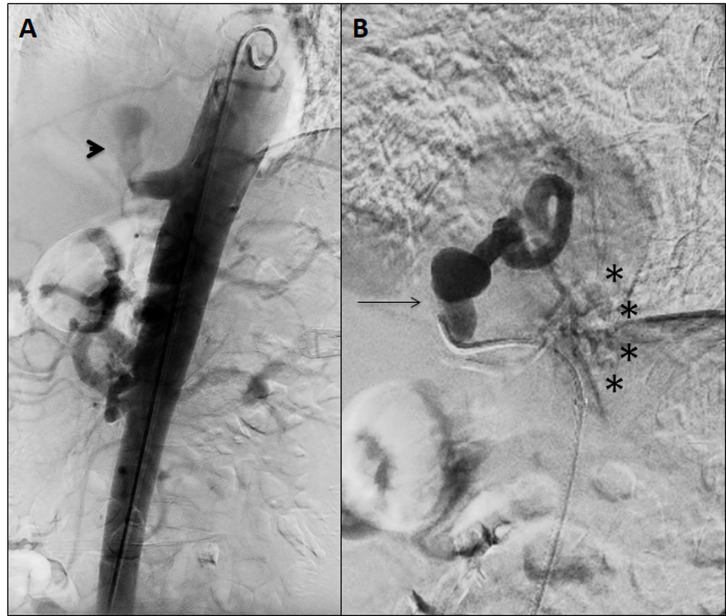
(A) Aberrant aneurysmal artery of PS on lateral angiography (arrowhead); (B) Selective angiogram showing the tortuous course of the aberrant artery (black arrow) terminating in the sequestration parenchyma (stars). *At this time, the contrast passed into the airway inducing cough.

**Figure 3:**
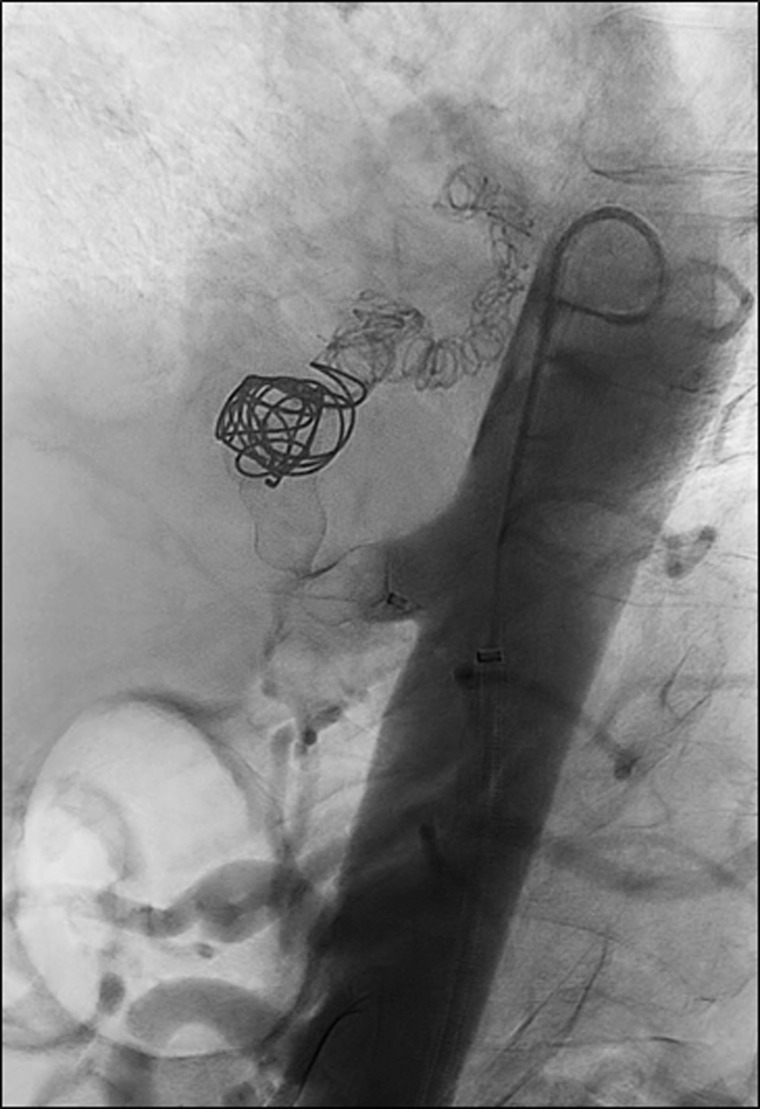
Post-embolization aortography shows the absence of flow after deployment of the Amplatzer II plug and coils along the aberrant aneurysmal artery.

Multiplanar aortography (Figure 2A) was performed through a 7 Fr Raabe® right femoral access (Cook Medical, Bloomington, IN, United States) under local anesthesia and endovascular treatment was performed after selective catheterization of the sequestration artery (Figure 2B). A selective angiography with a 5Fr glide catheter showed passage of contrast into the airway and induced cough (Figure 2B). The aneurysmal artery was then embolized with four 4-mm and four 15-mm Nester® coils (Cook Medical, Bloomington, IN, United States) at the distal end, six 16-mm Nester® coils (Cook Medical, Bloomington, IN, United States) in the aneurysmal zone, and a 14-mm Amplatzer Vascular Plug II® (AGA Medical, Plymouth, MN) was deployed in the proximal third of the sequestration artery. Angiography confirmed correct deployment, with no protrusion into the aorta and absence of flow in the sequestration artery (Figure 3). There were no incidents or complications during the procedure.

**Figure 4:**
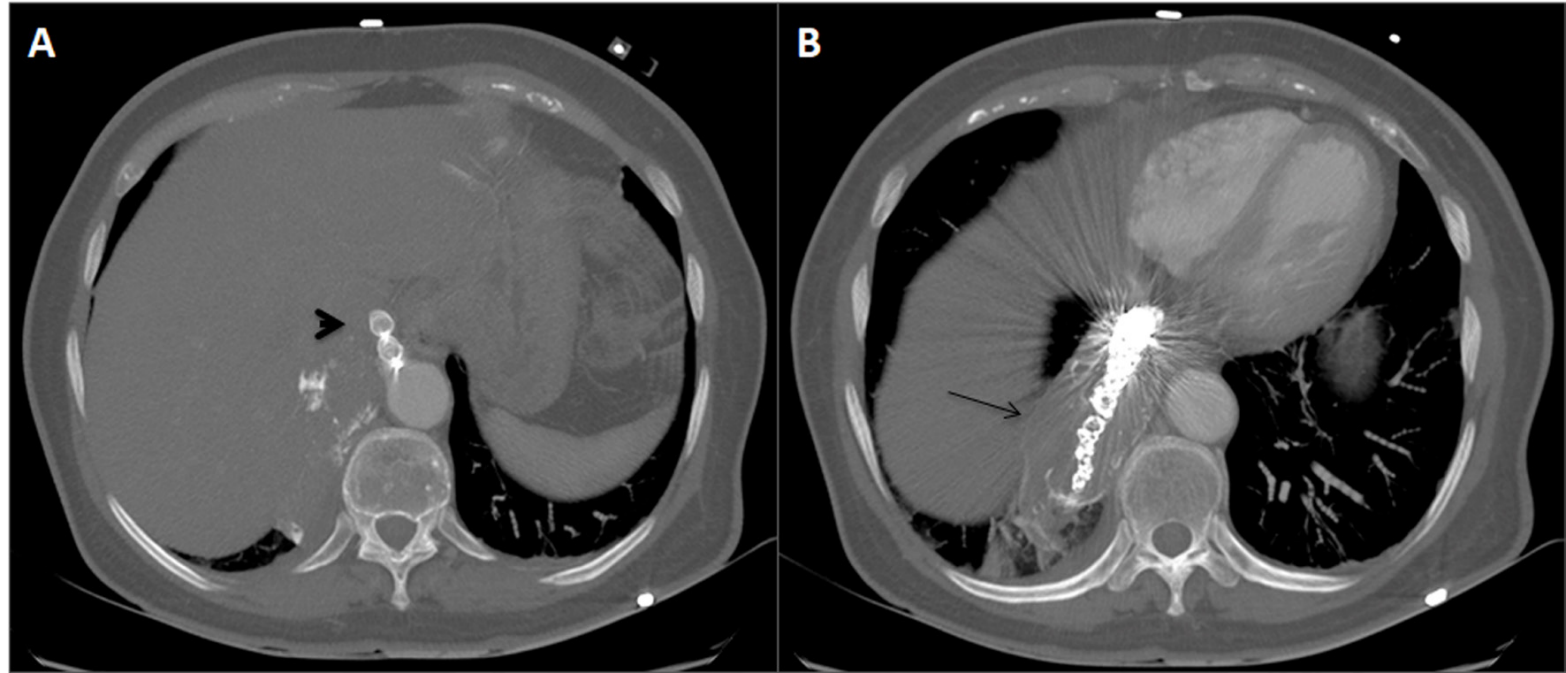
CTA performed before hospital discharge shows (A) the Amplatzer plug (arrowhead) and (B) the coils (black arrow), with absence of contrast and thrombus formation in the aneurysmal sac (black arrow in 4B).

In the following days, the patient was treated with ceftriaxone 2 g IV qd, acetaminophen 2 g PO q8h, heparin 5000 UI SC q12h, and chest physiotherapy twice daily. She had a good postoperative course, without hemoptysis, being discharged on postoperative day 4 with amoxicillin/clavulanate 875 mg/125 mg PO q12h for 3 days. Control CTA performed before hospital discharge confirmed complete thrombosis of the aberrant aneurysmal artery and absence of irrigation within the PS, without pleural effusion or signs of hemorrhage (Figure 4).

**Figure 5:**
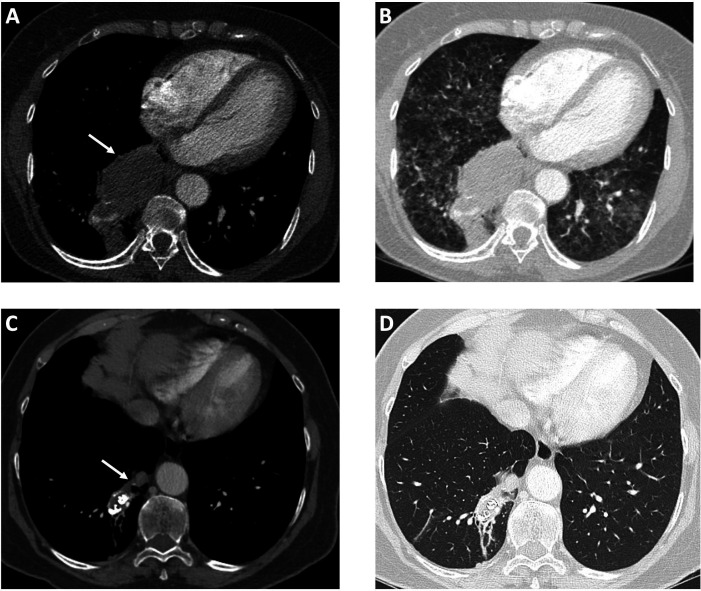
The figure shows a CTA performed at admission, before endovascular treatment (A) soft tissue window and (B) lung window; and a follow-up CTA performed 56 months after the intervention (C) soft tissue window and (D) lung window. A significant decrease in pulmonary sequestration size is observed, from 77 × 40 mm in 2016 to 28 × 18 mm in 2021 (white arrows in A and C).

At 56 months of follow-up, the patient remains asymptomatic, without recurrent hemoptysis. CTA at 1, 6, 16, and 56 months showed complete thrombosis of the aberrant artery and involution of the PS, with a reduction of 49 mm in its largest diameter (Figure 5).

## DISCUSSION

The presentation of PS is variable depending on whether it is intra or extralobar and on the number and origin of its feeding arteries. The blood supply to the PS originates most frequently from the descending thoracic aorta (46.1-86.1%).^2^ Origin from the abdominal aorta, as in the case presented, occurs less frequently (6.9-31.6%), and even less frequently from other aortic branches.^2^^,^^3^

Preoperative planning with CTA, MRI, or angiography allows adequate identification of the anatomy of the feeding arteries. This is essential to avoid intraoperative accidental injury, vessel retraction, and hemorrhagic death.^4^ A poor quality CTA may miss several of these arteries (20.9% of the cases) or those arising below the diaphragm.^5^

The arteries feeding the PS have some particularities that pose a challenge for treatment: they carry blood under systemic pressure, in a fragile, thin-walled artery, which can lead to accelerated atherosclerotic changes and is more susceptible to damage from recurrent pulmonary infections or tear injuries.^4^^,^^6^^,^^7^ In addition, inflammatory changes associated with recurrent PS infections distort the normal anatomy and can make open surgery very difficult.

Certain special considerations should be taken into account for treatment of the sequestration artery during open surgery or VATS. Liu et al. reported that iatrogenic vascular injury was the most common complication and cause of conversion in VATS.^3^ Zhang et al. reported that 2 of 15 cases of PS treated by thoracotomy lost more than 1,000 mL of blood during surgery.^8^ Fumimoto et al. described the technical maneuvers that should be additionally considered in VATS when stapling a sequestration artery of abdominal origin.^1^ The potential risks of massive hemorrhage or retraction into the abdomen make it advisable to completely resect the sequestration artery from its abdominal origin, requiring addition of laparotomy, lengthening the duration of surgery, or addition of embolization of its origin prior to pulmonary resection.^6^^,^^7^

Dilatation of the sequestration artery is extremely rare: Savic et al. reported one patient with an aneurysmal feeding artery in 540 PS reviewed,^9^ and Koyoma et al. found 2 in 21 Pryce type I intralobar PS.^10^ Surgical division of an aneurysmal feeding artery involves an increased risk of rupture during dissection or subsequent failure due to aneurysmal change of the ligation stump.^7^

Few cases of aneurysmal artery originating in the abdominal aorta have been reported.^7^^,^^10^^-^13 The natural history of sequestration artery aneurysm is unknown; it can potentially evolve with thrombosis or rupture. Kristensen et al. reported a similar case of PS and aneurysmal aberrant artery originating in the abdominal aorta that was treated with embolization.^11^ In that case, the patient consulted with back pain and recurrent pneumonia and the aneurysm was successfully embolized with an Amplatzer Vascular Plug II, without PS resection. The patient did not experience hemoptysis.

The case described in this report consulted with severe hemoptysis, hypotension, and significant drop in hematocrit, so it was necessary to stop the bleeding quickly and safely, taking into account the possible complications associated with a feeding artery that is aneurysmal and of abdominal origin. It was decided to embolize the entire length of the aneurysmal artery with coils and a vascular plug. Using an Amplatzer Vascular Plug II at the origin of the artery has the advantage of precision, since it can be recaptured if it prolapses into the aorta.

Feeding artery embolization as definitive treatment has been implemented for more than 20 years, but there is concern about damage to the lung during the procedure, incomplete treatment of the feeding arteries, and recurrence of symptoms, when open resection or VATS of the pathological lung will be required.^2^^,^^6^^,^^14^ To date, no treatment guidelines have been established. Zhang et al.^8^ retrospectively compared surgery and endovascular treatment in 28 patients and concluded that embolization could be considered when the pulmonary lesion is small in size (< 3 cm), whereas endovascular stent-graft exclusion could be used to treat aneurysm and dissection of the aberrant artery. One disadvantage of this therapeutic option is the lack of a pathology specimen to complete characterization of the PS.

We have previously published a case of PS with multiple feeding arteries that was treated exclusively with coil embolization and presented recurrent infection 7 months later. Repeated angiography showed persistence of a small feeding branch that was successfully treated with a new coil embolization, resulting in complete infarction of the sequestration.^15^ In the present new PS case, the single feeding artery was embolized completely and the patient remains asymptomatic 56 months after treatment.

In conclusion, endovascular treatment allows effective and minimally invasive management in selected cases of PS. In the present case, a symptomatic PS with a single aneurysmal abdominal feeding artery was successfully treated by embolization.
